# Differential gene expression in multiple neurological, inflammatory and connective tissue pathways in a spontaneous model of human small vessel stroke

**DOI:** 10.1111/nan.12116

**Published:** 2014-11-17

**Authors:** Emma L Bailey, Martin W McBride, Wendy Beattie, John D McClure, Delyth Graham, Anna F Dominiczak, Cathie LM Sudlow, Colin Smith, Joanna M Wardlaw

**Affiliations:** * Centre for Clinical Brain Sciences, University of Edinburgh, Western General HospitalEdinburgh; † Department of Bioengineering, Imperial College LondonLondon; ‡ Institute of Cardiovascular and Medical Sciences, University of GlasgowGlasgow; § Centre for Molecular Medicine, Institute of Genetics and Molecular Medicine, University of Edinburgh, Western General HospitalEdinburgh; ¶ Academic Department of Neuropathology, Centre for Clinical Brain Sciences, University of EdinburghEdinburgh; ** SINAPSE Collaboration (Scottish Imaging Network, A Platform for Scientific Excellence http://www.sinapse.ac.uk)Edinburgh, UK

**Keywords:** blood brain barrier, lacunar stroke, neurovascular unit, small vessel disease, stroke

## Abstract

**Aims:**

Cerebral small vessel disease (SVD) causes a fifth of all strokes plus diffuse brain damage leading to cognitive decline, physical disabilities and dementia. The aetiology and pathogenesis of SVD are unknown, but largely attributed to hypertension or microatheroma.

**Methods:**

We used the spontaneously hypertensive stroke-prone rat (SHRSP), the closest spontaneous experimental model of human SVD, and age-matched control rats kept under identical, non-salt-loaded conditions, to perform a blinded analysis of mRNA microarray, qRT-PCR and pathway analysis in two brain regions (frontal and mid-coronal) commonly affected by SVD in the SHRSP at age five, 16 and 21 weeks.

**Results:**

We found gene expression abnormalities, with fold changes ranging from 2.5 to 59 for the 10 most differentially expressed genes, related to endothelial tight junctions (reduced), nitric oxide bioavailability (reduced), myelination (impaired), glial and microglial activity (increased), matrix proteins (impaired), vascular reactivity (impaired) and albumin (reduced), consistent with protein expression defects in the same rats. All were present at age 5 weeks thus predating blood pressure elevation. ‘Neurological’ and ‘inflammatory’ pathways were more affected than ‘vascular’ functional pathways.

**Conclusions:**

This set of defects, although individually modest, when acting in combination could explain the SHRSP's susceptibility to microvascular and brain injury, compared with control rats. Similar combined, individually modest, but multiple neurovascular unit defects, could explain susceptibility to spontaneous human SVD.

## Introduction

Stroke is the second commonest cause of death and commonest cause of dependence in adults worldwide. A fifth of all strokes, a quarter of all ischaemic strokes, are lacunar in type and part of the spectrum of cerebral small vessel disease (SVD) [1]. SVD also causes cognitive and physical disabilities [2], doubles the risk of dementia and trebles the risk of stroke [3].

Cerebral SVD affects the penetrating arterioles supplying the cerebral white and deep grey matter [1]. Human pathology, often late stage and thus hard to unravel [4], includes arteriolar vessel wall thickening, perivascular inflammation, arteriolar wall disintegration [5] and perivascular damage particularly oedema and demyelination [6] commonly considered to be a consequence of ischaemia [4]. Hypertension is the main known vascular risk factor for SVD [7]. However, some individuals with pathological evidence of SVD lacked evidence of having been hypertensive in life [8], and trials of antihypertensive drugs have had mixed success in preventing SVD progression 9–11.

We considered that examination of different potential SVD mechanisms in experimental models might provide insight into pathogenesis of human SVD. Although several models exist we focused on the spontaneously hypertensive stroke-prone rat (SHRSP), which is genetically stable and known to mimic spontaneously the human microvascular and brain tissue changes [12],[13]. The SHRSP was bred from the Wistar-Kyoto rat (WKY) via the spontaneously hypertensive rat (SHR) [14]. It starts to develop hypertension after 6 weeks of age and strokes begin to occur from around 20 weeks [12]. It develops lipohyalinosis and fibrinoid necrosis, small deep infarcts, haemorrhages, white matter abnormalities and perivascular space enlargement spontaneously that resemble the pathological changes associated with human SVD [12],[14]. Although traditionally attributed to hypertension, we found differences in protein expression in SHRSPs (*vs.* age-matched WKY controls) at only 5 weeks of age, that is before blood pressure rises, which persist at 16 and 21 weeks of age [15]. These endothelial, glial, astrocyte, microglial and matrix protein abnormalities provide a potential mechanism to explain the SHRSP's vulnerability to cerebral microvascular and parenchymal damage [15].

Components of human SVD are highly heritable, but so far human genome-wide association studies (GWAS) have identified few genes associated with white matter hyperintensities (mostly of unknown function) and none so far for lacunar stroke 16–18. The genetic factors that render the SHRSP stroke-prone are also poorly understood. Known differences between SHRSP and WKY include STR-2 quantitative trait loci on chromosome 5 colocalized with genes encoding atrial and brain natriuretic peptides 19–21, a single-nucleotide polymorphism (SNP) on chromosome 2 (R202H) associated with reduced glutathione S-transferase expression [22], and, from a recent GWAS, a number of candidate genes for hypertension in spontaneously hypertensive rat substrains [23], but which are not known to affect stroke directly [24].

We hypothesized that the predisposition of SHRSP to cerebral microvascular and brain tissue pathology is multifactorial, involves several pathways, with no single genetic defect accounting for all the structural and pathological changes. We assessed mRNA expression in the brain by microarray, quantitative reverse transcriptase polymerase chain reaction (qRT-PCR) and pathway analysis in SHRSP and WKY fed a normal diet at 5, 16 and 21 weeks in the two brain regions (frontal and mid-coronal), which are known to express maximal pathology [15].

## Materials and methods

### Animals and tissue

All animals were kept and experiments conducted according to UK regulations for live animal research in licensed laboratories (licence No. 60/3618) and conducted according to the ARRIVE (Animal Research: Reporting In Vivo Experiments) guidelines (http://www.nc3rs.org/ARRIVE). We also report our results according to the Minimum Information About a Microarray Experiment (MIAME) 2.0 criteria (http://www.mged.org/Workgroups/MIAME/miame_2.0.html).

SHRSP rats were considered the most appropriate model of human SVD available based on a detailed systematic review undertaken within the lab [12]. It is accepted that no animal model will ever truly and completely represent a human disease, but the data generated from a carefully chosen model can inform and focus subsequent translational research.

We used four male SHRSP_Gla_ (designated SHRSP) and WKY_Gla_ (designated WKY) animals in each of three age groups (5, 16 and 21 weeks, total animals *n* = 24) from the Glasgow colony, the same animals having been used to generate earlier immunohistochemistry data, thereby providing a direct intra-animal immunohistochemistry-mRNA comparison. Five-week-old animals were considered prehypertensive as SHRSP have consistently not shown any difference in blood pressure (BP) until after 6 weeks in SHRSP from this colony in prior work compared with control rats [25], with BP measurements always taken from conscious animals. We considered 15-week-old rats to represent established hypertension, and 21 weeks as an age at which strokes were commonly seen to start. We kept all animals in identical conditions and fed both strains on standard rat chow *ad libitum* (Rat and Mouse No. 1 Maintenance Diet, Special Diet Services). We used tail cuff plethysmography to take measurements of systolic blood pressure on a weekly basis in older rats. We sacrificed animals by overdose of isofluorane anaesthetic plus exsanguination. We preserved one cerebral hemisphere in formalin and the other hemisphere in liquid nitrogen for mRNA analysis as previously described [15]. For mRNA analysis, we immersed each frozen hemisphere in ×10 volume of RNA-later ice solution and incubated for 24 h at −20°C so that the following day 2 mm coronal slices from a frontal and a mid-coronal region could be taken using a Zivic® rat slicer matrix (Zivic Instruments, Pittsburgh, PA, USA) which captured the frontal cortex, thalamus, internal capsule and basal ganglia. These represent the areas most susceptible to cerebral and vascular pathology in the SHRSP [12].

### RNA extraction

We placed each coronal slice in approximately 1.5 ml (×10 volume) of lysis buffer (Qiazol) and homogenized the tissue using a POLYTRON® homogenizer (Capitol Scientific Inc., Austin, TX, USA). We extracted RNA using a Qiagen RNAeasy lipid tissue minikit (Qiagen Ltd., Manchester, UK). We eluted the resulting RNA with nuclease free water (2 × 50 μl). We treated half the elute with turbo DNase to remove any remaining genomic DNA and assessed the quality of the resulting RNA on a Nanodrop 1000 and Agilent® bioanalyser 2100 (Agilent, Santa Clara, CA, USA).

### RNA amplification and purification

We performed transcriptions from RNA to cRNA using an Ambion® Illumina® Total Prep RNA amplification kit (Applied Biosystems, Foster City, CA, USA). We generated first-strand cDNA with a first-strand master mix containing an oligo (dT) tagged with a phage T7 promotor and second-strand cDNA with a master mix containing DNA polymerase. We added the *in vitro* master mix solution to the elute according to kit instructions to amplify and label the cDNA with biotin UTP. We obtained a final cRNA elute of ∼200 μl and checked cRNA quality on the Agilent® bioanalyser.

### Microarray mRNA expression analysis

We added 5 μl of cRNA (∼750 ng) to 10 μl of hybridization buffer and loaded the resulting 15 μl onto a RatRef12 microarray chip (Illumina, San Diego, CA, USA), containing 22 519 gene and probe sets. We incubated chips at 58°C overnight, washed them with E1BC solution and stained them in a solution of E1 buffer plus 1:1000 dilution of streptavidin-Cy3. We scanned chips on an Illumina® Bead Reader (Illumina, San Diego, CA, USA) and recorded the intensity of fluorescent signal emitted. Samples with a signal intensity of >600 passed the bead array reader's quality control. We randomized samples throughout the entire microarray protocol and all samples were hybridized to the chips and scanned at the same time.

### qRT-PCR

Using the same DNase-treated RNA from the microarray experiment as the template for the synthesis of cDNA, we performed qRT-PCR reactions using Applied Biosystems Taqman® Gene Expression Assay (Applied Biosystems, Foster City, CA, USA) to quantitatively confirm differential expression measured in the microarray experiment. Briefly, we reverse transcribed 20 μl reactions on a 96-well plate containing approximately 1 μg of RNA using the Taqman® reverse transcription master mix (Applied Biosystems, Foster City, CA, USA) including OligodT primers and Multiscribe™ reverse transcriptase enzyme. We performed qRT-PCR on the cDNA by creating a reaction mix in the same Eppendorf containing Taqman® universal master mix (Applied Biosystems, Foster City, CA, USA) plus a probe for a housekeeping (control) gene [Glyceraldehyde 3-phosphate dehydrogenase (GAPDH) (VIC® labelled)] and the Taqman® probe corresponding to our gene of interest (FAM® labelled). We ran samples on a 384-well plate on a 7900HT Sequence detector (Applied Biosystems, Foster City, CA, USA).

### Standard PCR

We designed forward and reverse primers corresponding to the portion of the GUCY1a3 gene sequence covered by the Illumina® microarray probe. To test for insertions or deletions within the sequence between strains we ran end-point PCR using these primers, on DNA taken from the livers of additional SHRSP (*n* = 4) and WKY (*n* = 3). We used ‘Kod Hot Start’ DNA polymerase (Novagen, Merck, Darmstadt, Germany) and performed PCR reactions using the designated kit and to manufacturer's instructions. Extension times depended on the length of sequence being created. We performed all PCR reactions on a MJ Research Peltier Thermal Cycler (http://www.mj-research.com) using 96-well plates. We analysed results using agarose gel electrophoresis. We visualized gels on a Bio-Rad Fluor-S Multimager and assessed the size of PCR products using Promega 100 bp or 1 kb DNA ladders (Promega, Madison, WI, USA).

### DNA sequencing

We used Applied Biosystems BigDye Terminator n3.1 Cycle Sequencing kits (Applied Biosystems, Foster City, CA, USA) for all sequencing reactions and performed reactions in 96-well plates (SHRSP *n* = 2, WKY *n* = 3 taken from the samples used for standard PCR). We loaded a reaction solution of sequencing buffer, ready reaction, primer, purified PCR product and water into each well. To sequence, we used a temperature cycling programme of 96°C for 45 s, 50°C for 25 s and 60°C for 4 min, repeated 25 times. We performed sequencing capillary electrophoresis on a 48-capillary Applied Biosystems 3730 Genetic Analyser with 36 cm capillaries filled with POP-7 polymer (Applied Biosystems, Foster City, CA, USA) and warmed to 60°C. We separated sequencing products by size using electrophoresis set to 8500 V for 50 min.

### Data analysis

#### Microarray data

We analysed data using Rank Products (RP) analysis, a nonparametric statistical technique [26] complete with Bejamini-Hochberg false discovery rate (FDR) adjustment for multiple testing. FDR < 0.05 was considered significant. We did not set a minimum individual fold change for significance as we were interested in pathway interactions. We first generated Venn diagrams to visualize the results by age and brain section, then uploaded focus genes onto the Ingenuity Pathway Analysis® (IPA) (Ingenuity Systems, http://www.ingenuity.com) and analysed data using both a prespecified candidate gene approach (looking for changes in genes and pathways thought to be relevant from previous work by ourselves and others) and a genome-wide approach (to generate new hypotheses). Significance of pathways was assessed using one-sided Fisher's exact tests.

#### qRT-PCR data

we exported cycle threshold (CT) values from sequence detection system (SDS) software into a Microsoft® Excel spreadsheet and compared the mean delta cycle threshold (dCT) values *vs.* the housekeeper gene (Student's *t*-test). From these values, we calculated and plotted the delta dCT (ddCT) and relative quantification of mRNA expression changes (2^∧^ddCT) values.

#### Sequencing data

We analysed sequencing using Applied Biosystems SeqScape® software and aligned experimental sequences with known sequences derived from bioinformatic databases such as ENSEMBL genome browser (http://www.ensembl.org/index.html).

## Results

At 5 weeks (prior to any rise in blood pressure), there were more differentially expressed genes between SHRSP and WKY than at 16 or 21 weeks: 162 were differentially expressed in both brain regions, plus 202 just in frontal and 88 just in mid-coronal sections (total 452). There were far fewer differentially expressed genes at 16 weeks (71 genes in both regions, 30 in frontal only, 20 in mid-coronal only, total 121) or 21 weeks (63 genes in both regions, 131 in frontal only, 47 in mid-coronal only, total 241).

**Figure 1:**
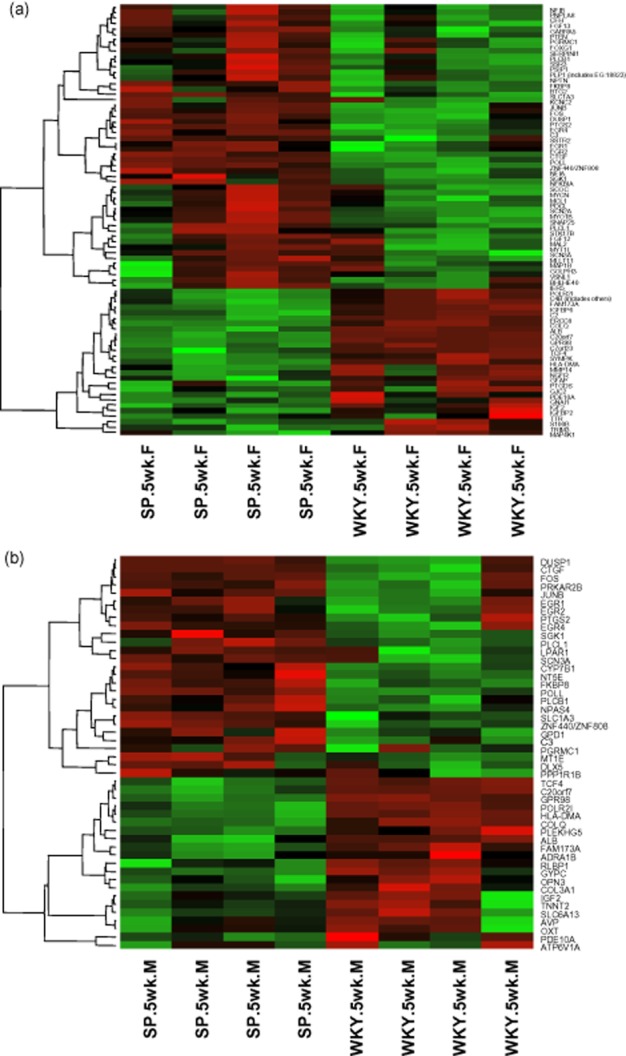
Heat maps were generated using hierarchical clustering of all significantly differentially expressed Neurological Genes from individual biological replicates of 5-week-old SHRSP and WKY animals. The relative expression of each gene is represented by colour intensity, where red is up and green is down-regulated in (a) 79 genes from the frontal region and (b) 48 genes identified in the mid-coronal region.

Ingenuity Pathway Analysis of all 452 differentially expressed genes at 5 weeks showed that these involved several functional pathways (Table 1), many of which remained affected at 16 and 21 weeks (Table 2). A common feature in both brain regions was the striking over-representation of genes for ‘neurological disease’ (Figure 1) particularly those involved in *encephalopathy*, *stroke*, *depression* and *blood brain barrier leakage* (Table 3; further details in Tables S1 and S2).

**Table 1:** Summary of analysis of differential gene expression by functional group in frontal and mid-coronal sections of 5-week-old SHRSP and WKY rats performed using Ingenuity Pathway Analysis (see Table S1 for similar analysis in 16- and 21-week rats)

	Frontal		Mid-coronal	
Number of molecules	*P*-value	Number of molecules	*P*-value
Diseases and disorders				
Neurological disease	79	1.51E-10–1.44E-02	48	3.80E-07–1.02E-02
Genetic disorder	84	1.92E-09–1.44E-02	41	6.19E-06–1.02E-02
Skeletal and muscular disorders	72	1.92E-09–1.44E-02	50	6.19E-06–1.02E-02
Connective tissue disorders	40	3.82E-06–1.44E-02	NA	NA
Inflammatory disease	48	3.82E-06–1.44E-02	NA	NA
Developmental disorder	NA	NA	34	1.12E-05–1.02E-02
Organismal injury and abnormalities	NA	NA	18	5.62E-05–8.69E-03
Molecular and cellular functions				
Gene expression	70	7.55E-07–1.44E-02	54	2.83E-08–1.02E-02
Cell death	86	5.00E-06–1.44E-02	NA	NA
Cell morphology	60	2.14E-05–1.44E-02	NA	NA
Carbohydrate metabolism	3	2.90E-05–1.44E-02	26	1.04E-06–1.02E-02
Lipid metabolism	21	2.90E-05–1.44E-02	54	5.18E-08–1.02E-02
Small molecule biochemistry	NA	NA	44	5.18E-08–1.02E-02
Cellular growth and proliferation	NA	NA	66	1.44E-06–1.02E-02

**Table 2:** Summary of differential gene expression by functional group in frontal and mid-coronal sections of 16- and 21-week-old SHRSP and WKY rats, performed using Ingenuity Pathway Analysis (see Table 1 for similar analysis of differentially expressed genes at 5 weeks)

	Frontal		Mid-coronal	
Number of molecules	*P*-value	Number of molecules	*P*-value
**16 weeks**				
Diseases and disorders				
Inflammatory response	4	7.68E-05–4.99E-02	6	7.68E-05–2.86E-02
Neurological disease	22	1.01E-03–4.61E-02	18	5.06E-05–3.91E-02
Connective tissue disorders	12	1.77E-03–2.86E-02	NA	NA
Inflammatory disease	15	1.77E-03–4.99E-02	NA	NA
Skeletal and muscular disorders	14	1.77E-03–2.86E-02	NA	NA
Psychological disorders	NA	NA	8	5.06E-05–7.23E-03
Infectious disease	NA	NA	7	1.38E-04–2.86E-02
Cardiovascular disease	NA	NA	6	5.68E-04–1.89E-02
Molecular and cellular functions				
Amino acid metabolism	6	7.68E-05–4.61E-02	4	7.68E-05–3.56E-02
Drug metabolism	4	7.68E-05–2.12E-02	4	7.68E-05–3.56E-02
Molecular transport	11	7.68E-05–4.95E-02	17	7.68E-05–4.26E-02
Small molecule biochemistry	16	7.68E-05–4.95E-02	19	7.68E-05–4.61E-02
Cell death	22	3.10E-03–4.61E-02	NA	NA
Nucleic acid metabolism	NA	NA	6	3.19E-04–4.61E-02
**21 weeks**				
Diseases and disorders				
Neurological disease	39	5.83E-08–2.19E-02	31	2.49E-07–2.06E-02
Skeletal and muscular disorders	40	6.18E-07–2.19E-02	29	2.49E-07–1.29E-02
Hereditary disorder	34	7.50E-06–2.19E-02	27	2.49E-07–1.50E-02
Cardiovascular disease	15	1.61E-04–2.19E-02	NA	NA
Developmental disorder	19	3.20E-04–2.19E-02	14	1.13E-04–2.06E-02
Inflammatory response	NA	NA	10	1.01E-04–2.06E-02
Molecular and cellular functions				
Cell-to-cell signalling and interaction	28	5.52E-06–2.19E-02	NA	NA
Cell cycle	9	5.38E-05–1.68E-02	NA	NA
Cellular assembly and organization	28	5.38E-05–2.19E-02	NA	NA
Cellular function and maintenance	39	1.61E-04–2.12E-02	NA	NA
Cell death	45	3.40E-04–2.19E-02	NA	NA
Amino acid metabolism	NA	NA	6	7.98E-06–2.06E-02
Small molecule biochemistry	NA	NA	21	7.98E-06–2.41E-02
Cell signalling	NA	NA	11	5.79E-05–2.06E-02
Molecular transport	NA	NA	22	5.79E-05–2.41E-02
Vitamin and mineral metabolism	NA	NA	13	5.79E-05–2.18E-02

NA, not applicable.

**Table 3:** Details of some differentially expressed genes in the ‘neurological disorders’ functional pathway identified in IPA related to encephalopathy, major depression, stroke and blood brain barrier leakage. More details of neurological and inflammatory pathways affected are provided in Tables S1 and S2

Functions annotation	*P*-value	Predicted activation state	Regulation *z*-score	Molecules
Encephalopathy	1.51E-10		−0.706	ALB, BHLHE40, C20orf7, C3, C4B (includes others), CTGF, DUSP1, EGR1, EGR2, EGR4, FAM173A, FGF12, FGF13, FKBP8, FOS, FOXG1, GABRA5, GFAP, GOLPH3, GPR98, HLA-DMA, IER5, JUNB, KCNC2, MAL2, MAP1B, MAP4K1, MYO1B, MYT1L, NFIA, NFIB, NGFR, PDCL, PDE10A, PGRMC1, PLCB1, PLP1 (includes EG:18823), POLL, POLR2I, PTEN, PTGS2, S100B, SCN2A, SCN3A, SCOC, SERPINI1, SGK1, SLC1A3, SNAP25, SSR3, STK17B, VSNL1, ZNF440/ZNF808
Major depression	1.78E-03			BTG2, C7orf23, GABRA5, GFAP, IGFBP2, PDE10A, PSIP1, SYMPK, TTR
Stroke	1.95E-03			ALB, GABRA5, NGFR, PTGS2, S100B, SNAP25, VSNL1
Leakage of blood–brain barrier	2.01E-03			PTGS2, SERPINI1

**Figure 2:**
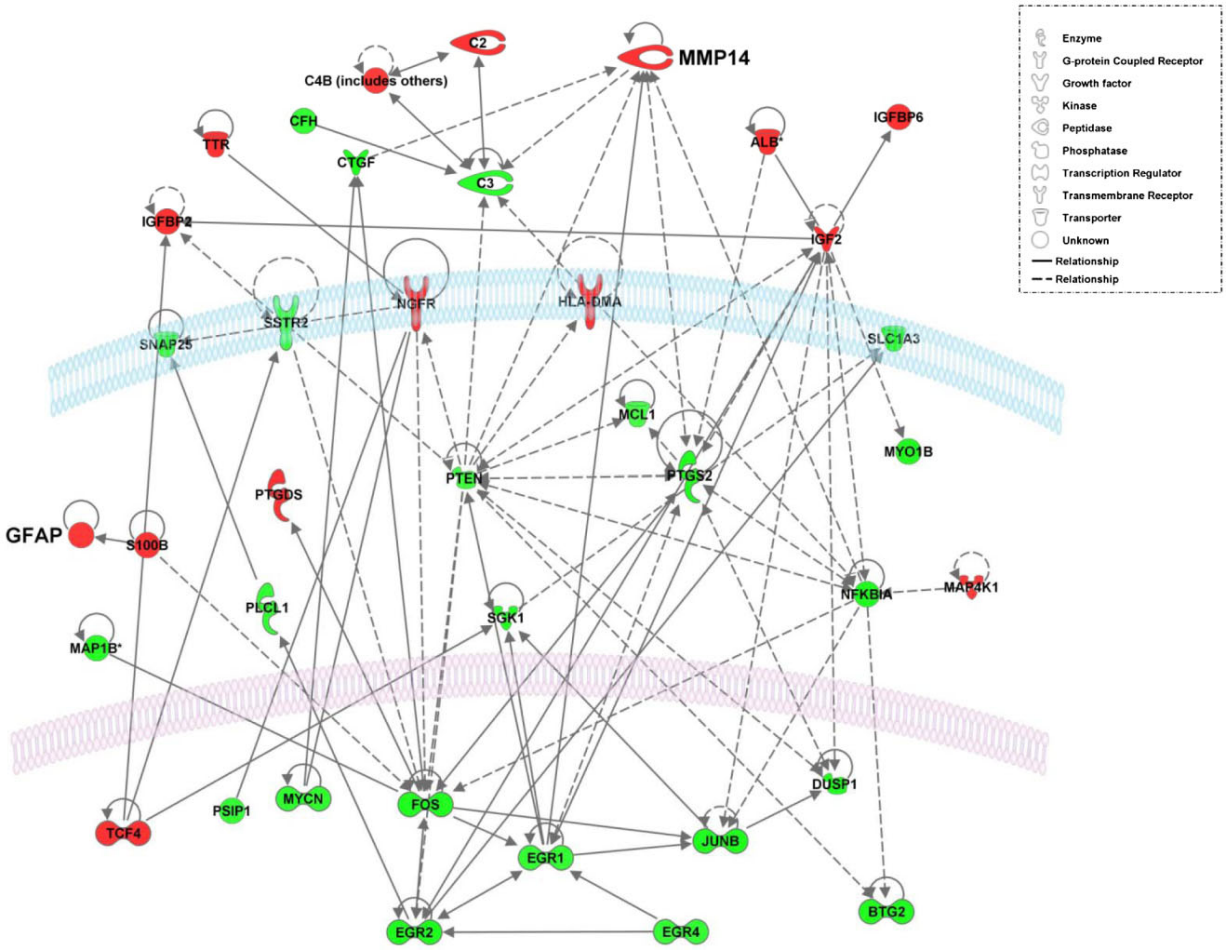
A network from the ‘neurological disorders’ functional group, representing interactions between differentially expressed genes in SHRSP *vs*. WKY rats at 5 weeks of age, generated by Ingenuity Pathway Analysis software. Genes highlighted in red are down-regulated and in green are up-regulated in SHRSP compared with WKY. Solid lines indicate direct interactions. Dotted lines indicate indirect interactions. Details of affected genes are given in Tables S1 and S2.

Network analysis of the differential gene expression showed significant up-regulation in SHRSP of transcription factors (Figure 2) including *Fos*, *JunB*, *Btg2* and early growth response genes (*Egr1*, *Egr2*, *Egr4*), genes central to cell signalling (*Pgs2* [*Cox2*], *Nfkbia*, *Pten*, *Sgk1*), and C3; others were down-regulated (e.g. insulin-like growth factors [*Igf2*], albumin [*Alb*], *Gfap* and *Mmp14*).

**Figure 3:**
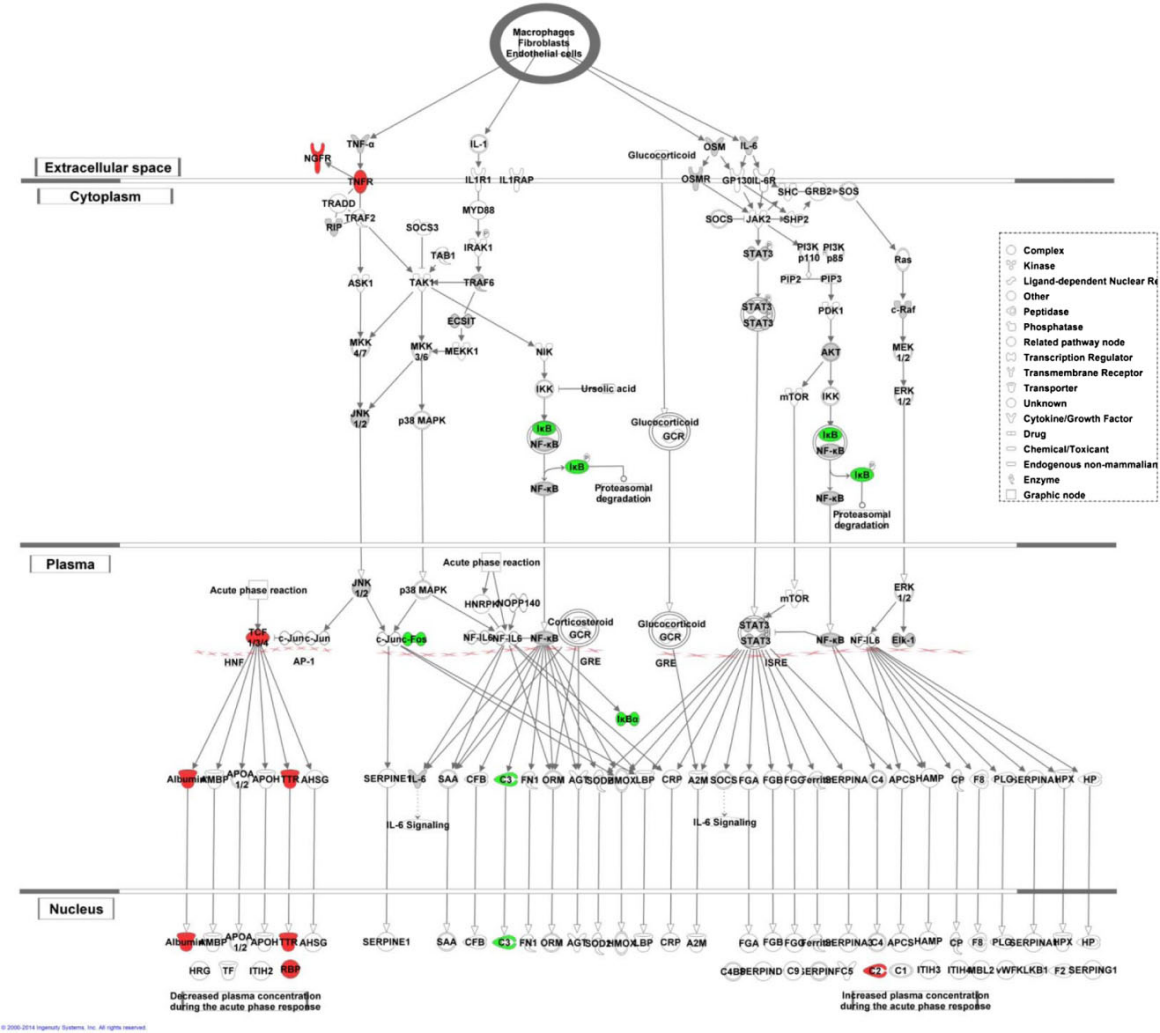
Details of genes in the ‘acute phase response’ biological pathway identified in Ingenuity Pathway Analysis, which are differentially expressed in the frontal section of SHRSP *vs.* WKY rats at 5 weeks of age. Genes highlighted red are down-regulated and in green are up-regulated in SHRSP compared with WKY. Solid lines indicate direct interactions. Dotted lines indicate indirect interactions.

**Figure 4:**
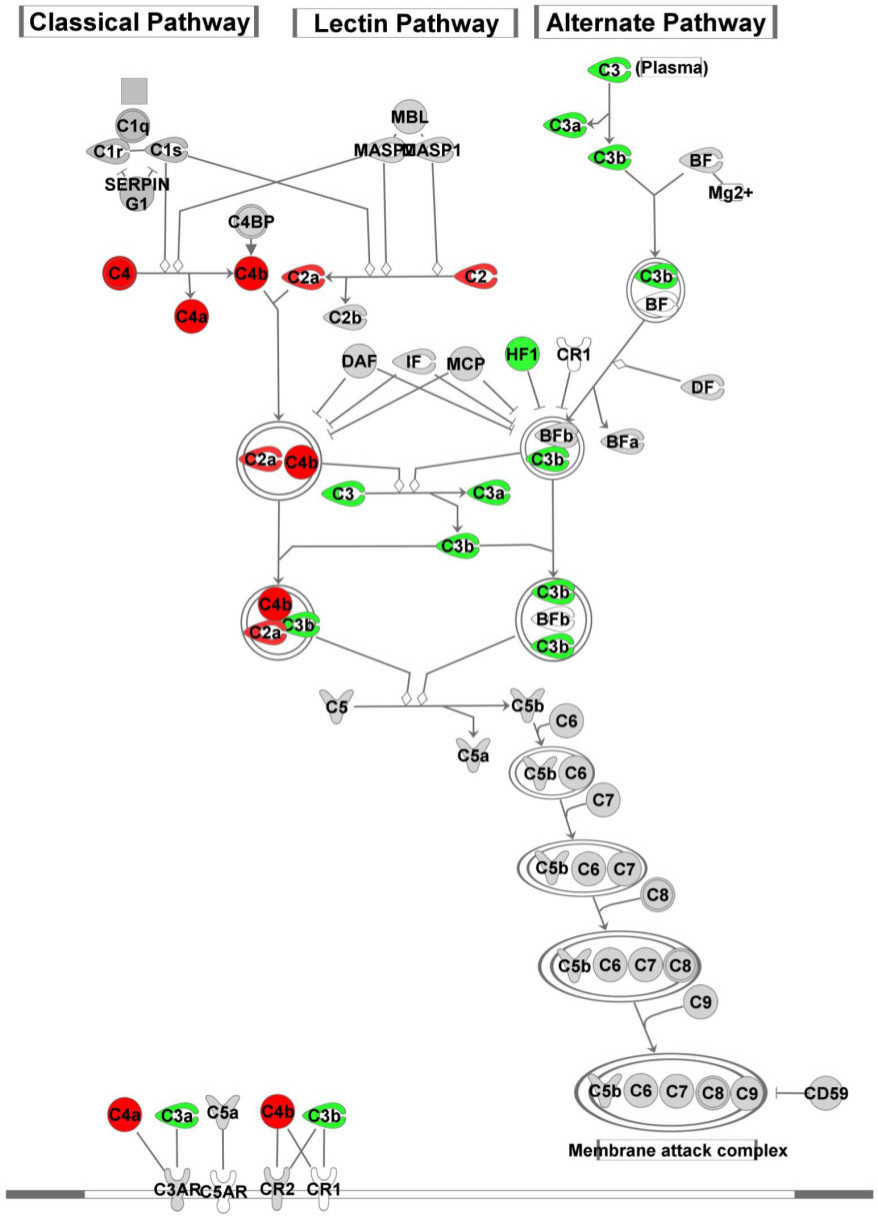
Details of genes in the ‘complement’ biological pathway identified in Ingenuity Pathway Analysis, which are differentially expressed in the frontal section of SHRSP *vs.* WKY rats at 5 weeks of age. Genes highlighted red are down-regulated and in green are up-regulated in SHRSP compared with WKY. Solid lines indicate direct interactions. Dotted lines indicate indirect interactions.

Across all ages, biological pathway analysis using IPA showed that the most significantly differentially expressed genes were in the acute phase response signalling pathway, but other pathways including circadian rhythm and complement were also affected (Figure 3). In the acute phase pathway, complement factor 3 (C3) was up-regulated, while *Alb* and *transcription factor 4* were down-regulated. All three components of the complement pathway (classic, lectin and alternate) were affected (*C3* up-regulated, *C2* and *C4* down-regulated) (Figure 4). The top five affected pathways also included genes associated with tight junction structure and signalling in 16- and 21-week-old SHRSP.

Among the most highly *up-regulated genes* were (Table 4): rat genome database (RGD) gene *1564649* related to urinary albumin secretion (up ∼48-fold); ribosomal protein S9 (*Rps9*) a translation regulator involved in cell proliferation (up ∼26-fold); and guanylate cyclase soluble subunit alpha-3 (*Gucy1a3*, up ∼20-fold), the intracellular nitric oxide (NO) vascular smooth muscle receptor. Among the most *down-regulated genes* were: *Alb* (down approximately threefold at all ages); arginine vasopressin (*Avp*, down fourfold at 5 weeks); guanine nucleotide-binding protein alpha inhibiting 1 (*Gnai1*, down threefold at 16 weeks); and *oxytocin* (down approximately threefold at 21 weeks).

**Table 4:** The top 10 up- and down-regulated genes in SHRSP *vs.* WKY in each brain section and for each age group. All genes listed are significantly differentially expressed when a FDR of *P* < 0.05 is applied

Age	Frontal section				Mid-coronal section			
	Up-regulated	Fold change	Down-regulated	Fold change	Up-regulated	Fold change	Down-regulated	Fold change
5	*RGD1564649*	×48.1	*Mrpl18*	×27.7	*RGD1564649*	×59.7	*Mrpl18*	×29.1
	*Rps9*	×26.4	*HCG 2004593*	×16.4	*Rps9*	×25.6	*HCG 2004593*	×13.2
	*Gucy1a3*	×21.8	*RGD1565336*	×5.3	*Gucy1a3*	×23.0	*RGD1565336*	×4.6
	*Fam151b*	×8.3	*Ttr*	×3.4	*Fam151B*	×6.7	*Avp*	×4.3
	*RGD1311103*	×4.9	*Gpr98*	×3.3	*RGD1311103*	×4.9	*LOC100125697*	×3.5
	*Arc*	×4.4	*LOC100125697*	×3.2	*Znf597*	×4.2	*Gpr98*	×3.2
	*Znf597*	×4.1	*Alb*	×3.1	*Rnf149*	×4.1	*Pxmp4*	×3.1
	*Rnf149*	×3.9	*Colq*	×2.9	*Arc*	×3.9	*Csnk2a1*	×2.6
	*Junb*	×3.5	*Pxmp4*	×2.8	*Dusp1*	×3.1	*Vps13c*	×2.6
	*Znf317*	×3.4	*HLA-C*	×2.6	*Rps16*	×3.0	*C20orf7*	×2.5
16	*RGD1564649*	×46.7	*Mrpl18*	×25.4	*RGD1564649*	×54.5	*Mrpl18*	×31.4
	*Rps9*	×24.0	*HCG 2004593*	×12.3	*Rps9*	×27.2	*HCG 2004593*	×12.9
	*Gucy1a3*	×19.8	*RGD1565336*	×4.7	*Gucy1a3*	×14.5	*RGD1565336*	×5.4
	*Fam151B*	×8.8	*LOC100125697*	×4.0	*Fam151b*	×7.9	*Gpr8*	×3.7
	*RGD1311103*	×5.4	*Pxmp4*	×3.9	*RGD1311103*	×6.6	*Alb*	×3.7
	*Znf597*	×4.0	*Alb*	×3.7	*Znf597*	×4.0	*LOC100125697*	×3.6
	*Rps16*	×3.1	*Gpr98*	×3.2	*Avp*	×3.8	*Vps13c*	×3.3
	*RGD1566136*	×2.8	*Gnai1*	×3.0	*RGD1566136*	×3.0	*Csnk2a1*	×3.3
	*HLA-C*	×2.8	*C7orf23*	×2.9	*Rsp16*	×2.9	*Pxmp4*	×3.1
	*Adpgk*	×2.7	*RGD1564078*	×2.8	*HLA-C*	×2.8	*C7orf23*	×3.0
21	*RGD1564649*	×46.9	*Mrpl18*	×30.7	*RGD1564649*	×45.6	*Mrpl18*	×25.9
	*Gucy1a3*	×20.3	*HCG 2004593*	×11.3	*Gucy1a3*	×20.7	*HCG 2004593*	×14.1
	*RSP9*	×18.8	*Ttr*	×7.4	*Rsp9*	×18.9	*RGD1565336*	×4.7
	*Fam151b*	×7.3	*RGD1565336*	×3.9	*Fam151b*	×7.3	*Gpr98*	×4.3
	*RGD1311103*	×6.6	*Alb*	×3.8	*RGD1311103*	×4.8	*Oxt*	×3.4
	*Znf597*	×4.8	*Gpr98*	×3.8	*Znf597*	×4.5	*Alb*	×3.3
	*RGD1566136*	×3.7	*Pxmp4*	×3.6	*Ttr*	×3.7	*Mobp*	×3.2
	*Rnf149*	×3.4	*Opcml*	×3.6	*Rnf149*	×3.4	*LOC100125697*	×3.1
	*Rps16*	×3.2	*Actb*	×3.4	*Adpgk*	×3.4	*Pxmp4*	×3.0
	*HLA-C*	×2.8	*LOC100125697*	×3.2	*HLA-C*	×3.3	*Vps13c*	×2.8

RGD, rat genome database; *Rps9*, ribosomal protein S9; *Gucy1a3*, guanylate cyclase 1, soluble, alpha 3; *Fam151b*, family with sequence similarity 151, member B; *Arc*, activity-regulated cytoskeleton-associated protein; *Znf*, zinc finger protein; *Rnf149*, ring finger protein 149; *Junb*, jun B proto-oncogene; *Mrpl18*, mitochondrial ribosomal protein L18; *Hcg*, human chorionic gonadotrophin; *Avp*, arginine vasopressin; LOC, location; *Gpr*, G-protein coupled receptor; *Alb*, albumin; *Colq*, collagen-like tail subunit of asymmetric acetylcholinesterase; *Pxmp4*, peroxisomal membrane protein 4; *Csnk2a1*, casein kinase 2, alpha 1 polypeptide; *Vps13c*, vacuolar protein sorting 13 homologue C; *C20orf7*, chromosome 20 open reading frame 7; *Hla-c*, myosin heavy chain class 1 receptor C; *Adpgk*, ADP-dependent glucokinase; *C7orf23*, chromosome 7 open reading frame 23; *Gnai1*, guanine nucleotide-binding protein, alpha inhibiting 1; *Opcml*, opioid-binding protein/cell adhesion molecule-like; *Actb*, beta actin; *Oxt*, oxytocin; *Mobp*, myelin-associated oligodendrocyte basic protein.

Compared with previously observed protein immunoreactivity [15], we saw no differences in mRNA expression of claudin-5, collagen I or Iba-1. However, *Mmp14* mRNA was down-regulated in SHRSP at 5 weeks and matrix metalloproteinase 14 (MMP14) has been shown to have a key role in modulating vessel stability and vascular responses to tissue injury by interacting with vascular molecules [27]. Down-regulated *Mbp* mRNA expression (2.6-fold) at 21 weeks was consistent with previously observed reduced myelin basic protein (MBP) immunoreactivity at all ages [15]. No relevant immunohistochemistry antibody was available to test for differential protein expression of AVP or GUCY1a3, but the mRNA data were consistent with other results 20–22.

**Figure 5:**
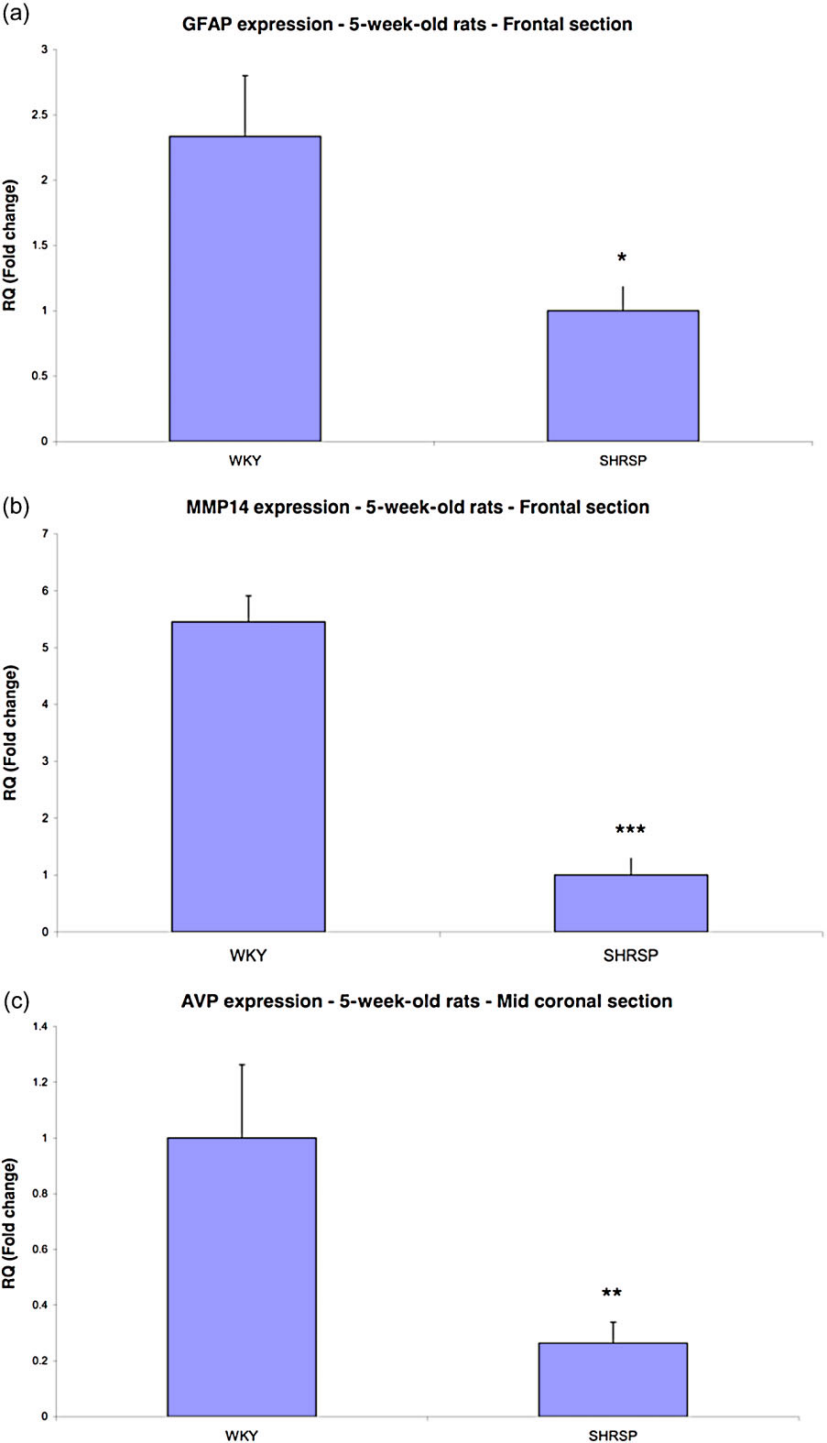
Validation of significant changes in gene expression of (a) GFAP, (b) MMP14 and (c) AVP in 5-week-old rats using qRT-PCR. Bars represent the difference in fold change between WKY and SHRSP. Error bars represent the standard error of the mean. Each bar represents *n* = 4 rats. *P < 0.05. **P < 0.01. ***P < 0.001. (a) GFAP mRNA expression was significantly reduced in the frontal section of SHRSP. (b) MMP14 mRNA expression was significantly reduced in the frontal section of SHRSP. (c) AVP mRNA expression was significantly reduced in the mid-coronal section of SHRSP.

qRT-PCR (Table S3) confirmed the microarray data for: MMP14 (down fivefold at 5 weeks, *P* < 0.01); GFAP (down twofold at 5 weeks, *P* = 0.01); and AVP (down fourfold at 5 weeks, *P* < 0.001) (Figure 5). qRT-PCR did not confirm the *Alb*, *Gucy1a3*, *Mbp* or *Gpr98* findings. The *Alb* difference may be a ‘floor effect’ where low mRNA expression, detectable by the greater dynamic range of the mRNA microarray, was undetectable by qRT-PCR. Despite consistent >15-fold *Gucy1a3* mRNA up-regulation in SHRSP at all ages in both brain sections, qRT-PCR showed no significant differences. Microarray and qRT-PCR probes examine different gene segments which are separated by >1000 nucleotides. Whole gene sequencing demonstrated a SNP in the 3′ untranslated region (UTR) of the *Gucy1a3* gene at position 4379 (WKY cytosine, SHRSP thymine). Discrepancies between mRNA microarray, qRT-PCR and immunohistochemistry could be further explained either by a true functional difference caused by microRNA binding to the 3′UTR region [28], or by post-translational modification [29]. Post-translational modification may be a result of over-exposure to a neurotransmitter or increased levels of reactive oxygen species [30] known to be present in the SHRSP [22].

## Discussion

Differential gene expression between SHRSP and WKY has been demonstrated, the top 10 differentially expressed genes having fold changes >2.5, and passing a FDR of *P* < 0.05. These differences were most pronounced at 5 weeks and diminished with age. The fact that the greatest difference in gene expression was seen in the youngest prehypertensive age group supports the hypothesis that there are genetic factors unrelated to primary hypertension that underlie susceptibility to damage to the neurovascular unit, and that some of these genes have reduced expression with ageing. It is possible that some of these pathways are related to age-specific processes such as developmental growth, although further genome analysis is required to assess this.

We demonstrate multiple transcription regulation differences across several key functional and biological pathways around the neurovascular unit that explain some of the SHRSP's complex brain pathology. The major biological pathways affected were ‘neurological’ and ‘inflammatory’. The most affected genes were generally conserved across all three ages examined. This constellation of differentially expressed genes may together increase vulnerability to microvascular damage and stroke. Possible mechanisms which may be involved, suggested by the data from this study and immunohistochemistry [15], include: impaired endothelial integrity (reduced claudin 5, MMP14) increasing the vascular endothelial permeability to plasma components which damage the arteriolar wall and subsequently the perivascular brain; reduced albumin facilitates fluid exudation into the vessel wall and brain interstitium through impaired plasma osmotic pressure [31]; microglial inflammatory responses are heightened, reducing NO bioavailability due to increased NO degradation from superoxide species 22,32–34; myelination (decreased MBP) is impaired and gliosis (increased GFAP) is increased, worsening brain damage; impaired vasoregulation (decreased *Avp* and *Gnai1*) and possibly reduced NO bioavailability (functional increase in NO receptor *Gucy1a3*) further increase vulnerability to ischaemia through impaired cerebral vasodilatation.

**Figure 6:**
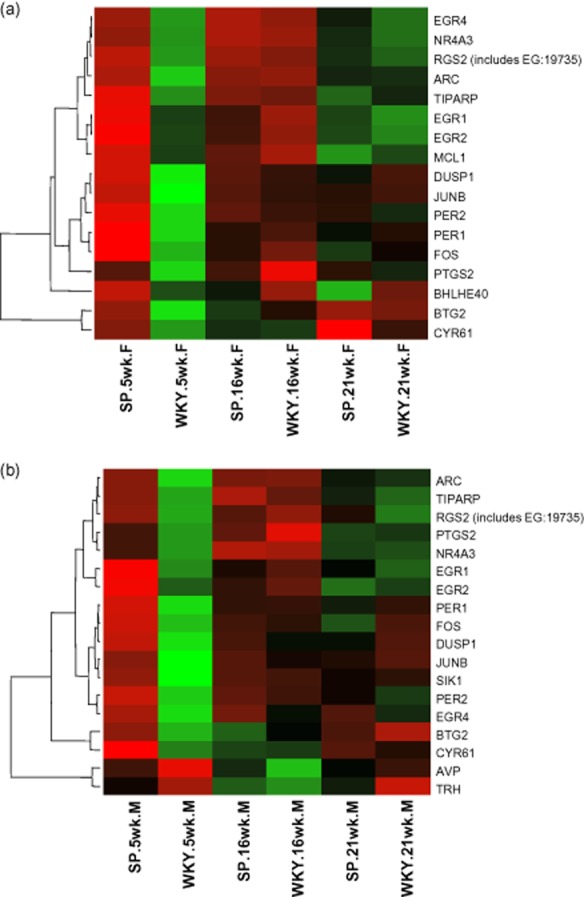
Heat maps generated using hierarchical clustering of the median expression from 5-, 16- and 21-week SHRSP and WKY downstream transcriptional targets of cAMP response element-binding protein (*Creb*). The relative expression of each probe is represented by colour intensity, where red is up and green is down-regulated in (a) 17 genes from the frontal (F) region and (b) 18 genes identified in the mid-coronal (M) region. Genes are listed on the right hand side.

Up-regulated inflammatory pathway genes indicate a chronically challenged immune system from early life also shown by others [12] which may trigger or accentuate the vascular and perivascular damage observed in older SHRSP compared with control strains. Several of the genes showing significant differential expression between SHRSP and WKY were downstream transcriptional targets of cAMP response element-binding protein (CREB) particularly the transcription factors (Figure 6). Genes regulated by CREB have been implicated in vascular remodelling in salt-induced hypertensive disease [35]. We found no difference in mRNA expression of *Creb1*, but differential phosphorylation of *Creb1*, initiated by NO [36] could be the source of increased transcription factor expression, and if shown in future experiments, would provide further evidence of a central role for altered intracellular NO signalling.

Reduced *Avp* is consistent with reported abnormalities in STR-2 loci [19] and associated with time to first stroke in SHRSP [20], highlighting the importance of intact vasoregulation. Potential functional differences in *Gucy1a3* are consistent with observed increases in endothelin 1A receptors [34] and lower bioavailability of NO in SHRSP due to increased NO degradation from superoxide species [22],[32],[33]. *Gucy1a3* was also identified in a recent GWAS of blood pressure and cardiovascular disease risk in humans [37].

The tight junction pathway defects concur with data from older SHRSP [38]. The strikingly and consistently abnormal acute phase response pathways concur with reports of proteinuria and raised serum inflammatory proteins prior to hypertension [39], although salt supplementation confounded these studies [12]. Increased complement and other inflammatory gene expression parallels the consistent observation of perivascular inflammation seen pathologically in humans for over a century [4]. Ten-month-old stroke-free SHRSP had *elevated* MBP in one study of rats from the same colony [40] in contrast to the present work. The rats in both studies were fed on the same diet and kept in identical conditions, so the different findings cannot be attributed to salt loading as in some earlier studies. The rats studied by Brittain *et al*. [40] at 10 months were the minority of stroke-free survivors as about 70% of the Glasgow SHRSP colony have had stroke by 9 months and died. As our rats were sacrificed young, we have no way of knowing if they were in the 70% that have stroke by 9 months or not. Of note, we did find histological evidence of early arteriolar fibrinoid microvascular changes in one SHRSP used in the gene expression analysis (EL Bailey and C Smith, unpub. obs.) in contrast to Brittain *et al*.

Although in this study we had limited numbers of rats, we had control animals kept under identical conditions, carefully blinded all analyses, had immunohistochemistry from the same rats' opposite hemispheres, and used multiple overlapping methods to check our findings.

We consider this SHRSP rat data to be valid animal model data to help focus translation research. In humans, stroke is a complex multifactorial disease, making a complex spontaneous disease model highly relevant. A large meta-analysis of stroke GWAS data by METASTROKE has emphasized the need for specific stroke subtyping and has strongly suggested that different pathogenic mechanisms underlie the different stroke subtypes [18]. The genetic architecture of most human cerebral small vessel stroke is unknown, hampered by imprecise stroke phenotyping and multiple accumulating ageing-related comorbidities. Brain microarray studies in elderly patients with dementia show multiple vessel wall, endothelial and brain parenchymal abnormalities including endothelial leakage [41] but precipitating factors and underlying susceptibility are difficult to unravel from end-stage disease. Human GWAS support the multifactorial nature of SVD [17]. Blood–brain barrier permeability is increased early in disease development in SHRSP [42], and is a key feature associated with human SVD in several primary studies [1]. Abnormal vasoregulation, long suspected in human SVD and associated with time to first stroke in SHRSP [19] could be the final factor that precipitates SVD brain lesions once the multifactorial milieu described above is established. Human data from the Leiden Longevity Study suggests that individuals who survive into late old age (equivalent to 10 months in SHRSP) are less susceptible to white matter damage and lacunar infarcts [43] and we would be interested to study older (10 months) stroke-free SHRSP rats to assess myelin integrity.

In summary, our data supports the multifactorial theory of SVD, with a number of genetic predispositions and biological pathways potentially contributing to the typical pathology. A similar targeted approach using new single cell mRNA technologies should be considered to determine if a similar pattern of low-level multifactorial defects in several components of the neurovascular unit might explain susceptibility to human SVD.

## Supporting Information

Table S1 Details of differentially expressed genes in the ‘neurological disorders’ functional pathway identified in IPA. Table S2. Details of differentially expressed genes in the ‘inflammation’ functional pathway identified in IPA. Table S3. Genes from the microarray chosen for quantitative validation with qRT-PCR, the reasons and data obtained from qRT-PCR analysis. Numbers are the mean difference (± the standard error of the mean) in CT values between the gene of interest and the house keeper gene GAPDH for SHRSP and WKY at ages 5–21 weeks and from frontal (F) and mid-coronal (M) brain sections.

## References

[1] Wardlaw JM, Smith C, Dichgans M (2013). Mechanisms of sporadic cerebral small vessel disease: insights from neuroimaging. Lancet Neurol.

[2] Baezner H, Blahak C, Poggesi A, Pantoni L, Inzitari D, Chabriat H, Erkinjuntti T, Fazekas F, Ferro JM, Langhorne P, O'Brien J, Scheltens P, Visser MC, Wahlund LO, Waldemar G, Wallin A, Hennerici MG (2008). Association of gait and balance disorders with age-related white matter changes: the LADIS study. Neurology.

[3] Debette S, Markus HS (2010). The clinical importance of white matter hyperintensities on brain magnetic resonance imaging: systematic review and meta-analysis. BMJ.

[4] Bailey EL, Smith C, Sudlow CL, Wardlaw JM (2012). Pathology of lacunar ischemic stroke in humans – a systematic review. Brain Pathol.

[5] Fisher CM (1968). The arterial lesions underlying lacunes. Acta Neuropathol.

[6] Fazekas F, Kleinert R, Offenbacher H, Schmidt R, Kleinert G, Payer F, Radner H, Lechner H (1993). Pathologic correlates of incidental MRI white matter signal hyperintensities. Neurology.

[7] Gottesman RF, Coresh J, Catellier DJ, Sharrett AR, Rose KM, Coker LH, Shibata DK, Knopman DS, Jack CR, Mosley TH (2010). Blood pressure and white-matter disease progression in a biethnic cohort: Atherosclerosis Risk in Communities (ARIC) study. Stroke.

[8] Lammie GA, Brannan F, Slattery J, Warlow C (1997). Nonhypertensive cerebral small-vessel disease. An autopsy study. Stroke.

[9] Benavente OR, Hart RG, McClure LA, Szychowski JM, Coffey CS, Pearce LA (2012). Effects of clopidogrel added to aspirin in patients with recent lacunar stroke. N Engl J Med.

[10] Dufouil C, Chalmers J, Coskun O, Besancon V, Bousser MG, Guillon P, MacMahon S, Mazoyer B, Neal B, Woodward M, Tzourio-Mazoyer N, Tzourio C (2005). Effects of blood pressure lowering on cerebral white matter hyperintensities in patients with stroke: the PROGRESS (Perindopril Protection Against Recurrent Stroke Study) Magnetic Resonance Imaging Substudy. Circulation.

[11] Weber R, Weimar C, Blatchford J, Hermansson K, Wanke I, Moller-Hartmann C, Gizewski ER, Forsting M, Demchuk AM, Sacco RL, Saver JL, Warach S, Diener HC, Diehl A (2012). Telmisartan on top of antihypertensive treatment does not prevent progression of cerebral white matter lesions in the prevention regimen for effectively avoiding second strokes (PRoFESS) MRI substudy. Stroke.

[12] Bailey EL, Smith C, Sudlow CL, Wardlaw JM (2011). Is the spontaneously hypertensive stroke prone rat a pertinent model of sub cortical ischemic stroke? A systematic review. Int J Stroke.

[13] Hainsworth AH, Markus HS (2008). Do in vivo experimental models reflect human cerebral small vessel disease? A systematic review. J Cereb Blood Flow Metab.

[14] Yamori Y, Horie R, Handa H, Sato M, Fukase M (1976). Pathogenetic similarity of strokes in stroke-prone spontaneously hypertensive rats and humans. Stroke.

[15] Bailey EL, Wardlaw JM, Graham D, Dominiczak AF, Sudlow CL, Smith C (2011). Cerebral small vessel endothelial structural changes predate hypertension in stroke-prone spontaneously hypertensive rats: a blinded, controlled immunohistochemical study of 5- to 21-week-old rats. Neuropathol Appl Neurobiol.

[16] Adib-Samii P, Rost N, Traylor M, Devan W, Biffi A, Lanfranconi S, Fitzpatrick K, Bevan S, Kanakis A, Valant V, Gschwendtner A, Malik R, Richie A, Gamble D, Segal H, Parati EA, Ciusani E, Holliday EG, Maguire J, Wardlaw J, Worrall B, Bis J, Wiggins KL, Longstreth W, Kittner SJ, Cheng YC, Mosley T, Falcone GJ, Furie KL, Leiva-Salinas C, Lau BC, Saleem Khan M, Sharma P, Fornage M, Mitchell BD, Psaty BM, Sudlow C, Levi C, Boncoraglio GB, Rothwell PM, Meschia J, Dichgans M, Rosand J, Markus HS (2013). 17q25 locus is associated with white matter hyperintensity volume in ischemic stroke, but not with lacunar stroke status. Stroke.

[17] Fornage M, Debette S, Bis JC, Schmidt H, Ikram MA, Dufouil C, Sigurdsson S, Lumley T, DeStefano AL, Fazekas F, Vrooman HA, Shibata DK, Maillard P, Zijdenbos A, Smith AV, Gudnason H, de Boer R, Cushman M, Mazoyer B, Heiss G, Vernooij MW, Enzinger C, Glazer NL, Beiser A, Knopman DS, Cavalieri M, Niessen WJ, Harris TB, Petrovic K, Lopez OL, Au R, Lambert JC, Hofman A, Gottesman RF, Garcia M, Heckbert SR, Atwood LD, Catellier DJ, Uitterlinden AG, Yang Q, Smith NL, Aspelund T, Romero JR, Rice K, Taylor KD, Nalls MA, Rotter JI, Sharrett R, van Duijn CM, Amouyel P, Wolf PA, Gudnason V, van der Lugt A, Boerwinkle E, Psaty BM, Seshadri S, Tzourio C, Breteler MM, Mosley TH, Schmidt R, Longstreth WT, DeCarli C, Launer LJ (2011). Genome-wide association studies of cerebral white matter lesion burden: the CHARGE consortium. Ann Neurol.

[18] Traylor M, Farrall M, Holliday EG, Sudlow C, Hopewell JC, Cheng YC, Fornage M, Ikram MA, Malik R, Bevan S, Thorsteinsdottir U, Nalls MA, Longstreth W, Wiggins KL, Yadav S, Parati EA, Destefano AL, Worrall BB, Kittner SJ, Khan MS, Reiner AP, Helgadottir A, Achterberg S, Fernandez-Cadenas I, Abboud S, Schmidt R, Walters M, Chen WM, Ringelstein EB, O'Donnell M, Ho WK, Pera J, Lemmens R, Norrving B, Higgins P, Benn M, Sale M, Kuhlenbaumer G, Doney AS, Vicente AM, Delavaran H, Algra A, Davies G, Oliveira SA, Palmer CN, Deary I, Schmidt H, Pandolfo M, Montaner J, Carty C, de Bakker PI, Kostulas K, Ferro JM, van Zuydam NR, Valdimarsson E, Nordestgaard BG, Lindgren A, Thijs V, Slowik A, Saleheen D, Pare G, Berger K, Thorleifsson G, Hofman A, Mosley TH, Mitchell BD, Furie K, Clarke R, Levi C, Seshadri S, Gschwendtner A, Boncoraglio GB, Sharma P, Bis JC, Gretarsdottir S, Psaty BM, Rothwell PM, Rosand J, Meschia JF, Stefansson K, Dichgans M, Markus HS (2012). Genetic risk factors for ischaemic stroke and its subtypes (the METASTROKE collaboration): a meta-analysis of genome-wide association studies. Lancet Neurol.

[19] Jeffs B, Clark JS, Anderson NH, Gratton J, Brosnan MJ, Gauguier D, Reid JL, Macrae IM, Dominiczak AF (1997). Sensitivity to cerebral ischaemic insult in a rat model of stroke is determined by a single genetic locus. Nat Genet.

[20] Rubattu S, Lee-Kirsch MA, DePaolis P, Giliberti R, Gigante B, Lombardi A, Volpe M, Lindpaintner K (1999). Altered structure, regulation, and function of the gene encoding the atrial natriuretic peptide in the stroke-prone spontaneously hypertensive rat. Circ Res.

[21] Rubattu S, Volpe M, Kreutz R, Ganten U, Ganten D, Lindpaintner K (1996). Chromosomal mapping of quantitative trait loci contributing to stroke in a rat model of complex human disease. Nat Genet.

[22] McBride MW, Brosnan MJ, Mathers J, McLellan LI, Miller WH, Graham D, Hanlon N, Hamilton CA, Polke JM, Lee WK, Dominiczak AF (2005). Reduction of Gstm1 expression in the stroke-prone spontaneously hypertension rat contributes to increased oxidative stress. Hypertension.

[23] Kinoshita K, Ashenagar MS, Tabuchi M, Higashino H (2011). Whole rat DNA array survey for candidate genes related to hypertension in kidneys from three spontaneously hypertensive rat substrains at two stages of age and with hypotensive induction caused by hydralazine hydrochloride. Exp Ther Med.

[24] Nabika T, Ohara H, Kato N, Isomura M (2012). The stroke-prone spontaneously hypertensive rat: still a useful model for post-GWAS genetic studies?. Hypertens Res.

[25] Koh-Tan HH, Graham D, Hamilton CA, Nicoll G, Fields L, McBride MW, Young B, Dominiczak AF (2009). Renal and vascular glutathione S-transferase mu is not affected by pharmacological intervention to reduce systolic blood pressure. J Hypertens.

[26] Breitling R, Armengaud P, Amtmann A, Herzyk P (2004). Rank products: a simple, yet powerful, new method to detect differentially regulated genes in replicated microarray experiments. FEBS Lett.

[27] Sounni NE, Dehne K, van Kempen L, Egeblad M, Affara NI, Cuevas I, Wiesen J, Junankar S, Korets L, Lee J, Shen J, Morrison CJ, Overall CM, Krane SM, Werb Z, Boudreau N, Coussens LM (2010). Stromal regulation of vessel stability by MMP14 and TGFbeta. Dis Model Mech.

[28] Nossent AY, Hansen JL, Doggen C, Quax PH, Sheikh SP, Rosendaal FR (2011). SNPs in microRNA binding sites in 3′-UTRs of RAAS genes influence arterial blood pressure and risk of myocardial infarction. Am J Hypertens.

[29] Etienne W, Meyer MH, Peppers J, Meyer RA (2004). Comparison of mRNA gene expression by RT-PCR and DNA microarray. Biotechniques.

[30] Adachi T (2010). Modulation of vascular sarco/endoplasmic reticulum calcium ATPase in cardiovascular pathophysiology. Adv Pharmacol.

[31] Menzies SA, Betz AL, Hoff JT (1993). Contributions of ions and albumin to the formation and resolution of ischemic brain edema. J Neurosurg.

[32] Gotoh K, Kikuchi H, Kataoka H, Nagata I, Nozaki K, Takahashi JC, Hazama F (1996). Altered nitric oxide synthase immunoreactivity in the brain of stroke-prone spontaneously hypertensive rats. Acta Neuropathol.

[33] Grunfeld S, Hamilton CA, Mesaros S, McClain SW, Dominiczak AF, Bohr DF, Malinski T (1995). Role of superoxide in the depressed nitric oxide production by the endothelium of genetically hypertensive rats. Hypertension.

[34] Uehara Y (2003). The world of endothelin in the brain of the stroke-prone spontaneously hypertensive rat. J Hypertens.

[35] Rose P, Bond J, Tighe S, Toth MJ, Wellman TL, Briso de Montiano EM, Lewinter MM, Lounsbury KM (2008). Genes overexpressed in cerebral arteries following salt-induced hypertensive disease are regulated by angiotensin II, JunB, and CREB. Am J Physiol Heart Circ Physiol.

[36] Riccio A, Alvania RS, Lonze BE, Ramanan N, Kim T, Huang Y, Dawson TM, Snyder SH, Ginty DD (2006). A nitric oxide signaling pathway controls CREB-mediated gene expression in neurons. Mol Cell.

[37] Ehret GB, Munroe PB, Rice KM, Bochud M, Johnson AD, Chasman DI, Smith AV, Tobin MD, Verwoert GC, Hwang SJ, Pihur V, Vollenweider P, O'Reilly PF, Amin N, Bragg-Gresham JL, Teumer A, Glazer NL, Launer L, Zhao JH, Aulchenko Y, Heath S, Sober S, Parsa A, Luan J, Arora P, Dehghan A, Zhang F, Lucas G, Hicks AA, Jackson AU, Peden JF, Tanaka T, Wild SH, Rudan I, Igl W, Milaneschi Y, Parker AN, Fava C, Chambers JC, Fox ER, Kumari M, Go MJ, van der Harst P, Kao WH, Sjogren M, Vinay DG, Alexander M, Tabara Y, Shaw-Hawkins S, Whincup PH, Liu Y, Shi G, Kuusisto J, Tayo B, Seielstad M, Sim X, Nguyen KD, Lehtimaki T, Matullo G, Wu Y, Gaunt TR, Onland-Moret NC, Cooper MN, Platou CG, Org E, Hardy R, Dahgam S, Palmen J, Vitart V, Braund PS, Kuznetsova T, Uiterwaal CS, Adeyemo A, Palmas W, Campbell H, Ludwig B, Tomaszewski M, Tzoulaki I, Palmer ND, Aspelund T, Garcia M, Chang YP, O'Connell JR, Steinle NI, Grobbee DE, Arking DE, Kardia SL, Morrison AC, Hernandez D, Najjar S, McArdle WL, Hadley D, Brown MJ, Connell JM, Hingorani AD, Day IN, Lawlor DA, Beilby JP, Lawrence RW, Clarke R, Hopewell JC, Ongen H, Dreisbach AW, Li Y, Young JH, Bis JC, Kahonen M, Viikari J, Adair LS, Lee NR, Chen MH, Olden M, Pattaro C, Bolton JA, Kottgen A, Bergmann S, Mooser V, Chaturvedi N, Frayling TM, Islam M, Jafar TH, Erdmann J, Kulkarni SR, Bornstein SR, Grassler J, Groop L, Voight BF, Kettunen J, Howard P, Taylor A, Guarrera S, Ricceri F, Emilsson V, Plump A, Barroso I, Khaw KT, Weder AB, Hunt SC, Sun YV, Bergman RN, Collins FS, Bonnycastle LL, Scott LJ, Stringham HM, Peltonen L, Perola M, Vartiainen E, Brand SM, Staessen JA, Wang TJ, Burton PR, Soler Artigas M, Dong Y, Snieder H, Wang X, Zhu H, Lohman KK, Rudock ME, Heckbert SR, Smith NL, Wiggins KL, Doumatey A, Shriner D, Veldre G, Viigimaa M, Kinra S, Prabhakaran D, Tripathy V, Langefeld CD, Rosengren A, Thelle DS, Corsi AM, Singleton A, Forrester T, Hilton G, McKenzie CA, Salako T, Iwai N, Kita Y, Ogihara T, Ohkubo T, Okamura T, Ueshima H, Umemura S, Eyheramendy S, Meitinger T, Wichmann HE, Cho YS, Kim HL, Lee JY, Scott J, Sehmi JS, Zhang W, Hedblad B, Nilsson P, Smith GD, Wong A, Narisu N, Stancakova A, Raffel LJ, Yao J, Kathiresan S, O'Donnell CJ, Schwartz SM, Ikram MA, Longstreth WT, Mosley TH, Seshadri S, Shrine NR, Wain LV, Morken MA, Swift AJ, Laitinen J, Prokopenko I, Zitting P, Cooper JA, Humphries SE, Danesh J, Rasheed A, Goel A, Hamsten A, Watkins H, Bakker SJ, van Gilst WH, Janipalli CS, Mani KR, Yajnik CS, Hofman A, Mattace-Raso FU, Oostra BA, Demirkan A, Isaacs A, Rivadeneira F, Lakatta EG, Orru M, Scuteri A, Ala-Korpela M, Kangas AJ, Lyytikainen LP, Soininen P, Tukiainen T, Wurtz P, Ong RT, Dorr M, Kroemer HK, Volker U, Volzke H, Galan P, Hercberg S, Lathrop M, Zelenika D, Deloukas P, Mangino M, Spector TD, Zhai G, Meschia JF, Nalls MA, Sharma P, Terzic J, Kumar MV, Denniff M, Zukowska-Szczechowska E, Wagenknecht LE, Fowkes FG, Charchar FJ, Schwarz PE, Hayward C, Guo X, Rotimi C, Bots ML, Brand E, Samani NJ, Polasek O, Talmud PJ, Nyberg F, Kuh D, Laan M, Hveem K, Palmer LJ, van der Schouw YT, Casas JP, Mohlke KL, Vineis P, Raitakari O, Ganesh SK, Wong TY, Tai ES, Cooper RS, Laakso M, Rao DC, Harris TB, Morris RW, Dominiczak AF, Kivimaki M, Marmot MG, Miki T, Saleheen D, Chandak GR, Coresh J, Navis G, Salomaa V, Han BG, Zhu X, Kooner JS, Melander O, Ridker PM, Bandinelli S, Gyllensten UB, Wright AF, Wilson JF, Ferrucci L, Farrall M, Tuomilehto J, Pramstaller PP, Elosua R, Soranzo N, Sijbrands EJ, Altshuler D, Loos RJ, Shuldiner AR, Gieger C, Meneton P, Uitterlinden AG, Wareham NJ, Gudnason V, Rotter JI, Rettig R, Uda M, Strachan DP, Witteman JC, Hartikainen AL, Beckmann JS, Boerwinkle E, Vasan RS, Boehnke M, Larson MG, Jarvelin MR, Psaty BM, Abecasis GR, Chakravarti A, Elliott P, van Duijn CM, Newton-Cheh C, Levy D, Caulfield MJ, Johnson T (2011). Genetic variants in novel pathways influence blood pressure and cardiovascular disease risk. Nature.

[38] Lippoldt A, Kniesel U, Liebner S, Kalbacher H, Kirsch T, Wolburg H, Haller H (2000). Structural alterations of tight junctions are associated with loss of polarity in stroke-prone spontaneously hypertensive rat blood-brain barrier endothelial cells. Brain Res.

[39] Sironi L, Tremoli E, Miller I, Guerrini U, Calvio AM, Eberini I, Gemeiner M, Asdente M, Paoletti R, Gianazza E (2001). Acute-phase proteins before cerebral ischemia in stroke-prone rats: identification by proteomics. Stroke.

[40] Brittain JF, McCabe C, Khatun H, Kaushal N, Bridges LR, Holmes WM, Barrick TR, Graham D, Dominiczak AF, Mhairi Macrae I, Hainsworth AH, An MRI (2013). histological study of white matter in stroke-free SHRSP. J Cereb Blood Flow Metab.

[41] Simpson JE, Hosny O, Wharton SB, Heath PR, Holden H, Fernando MS, Matthews F, Forster G, O'Brien JT, Barber R, Kalaria RN, Brayne C, Shaw PJ, Lewis CE, Ince PG (2009). Microarray RNA expression analysis of cerebral white matter lesions reveals changes in multiple functional pathways. Stroke.

[42] Sironi L, Guerrini U, Tremoli E, Miller I, Gelosa P, Lascialfari A, Zucca I, Eberini I, Gemeiner M, Paoletti R, Gianazza E (2004). Analysis of pathological events at the onset of brain damage in stroke-prone rats: a proteomics and magnetic resonance imaging approach. J Neurosci Res.

[43] Altmann-Schneider I, van der Grond J, Slagboom PE, Westendorp RG, Maier AB, van Buchem MA, de Craen AJ (2013). Lower susceptibility to cerebral small vessel disease in human familial longevity: the Leiden Longevity Study. Stroke.

