# Supplementary material for: Differential gene expression in multiple neurological, inflammatory and connective tissue pathways in a spontaneous model of human small vessel stroke
Source: Neuropathol Appl Neurobiol. 2014 Nov 17;40(7):855–72. doi: 10.1111/nan.12116 (PMC4260148; doi:10.1111/nan.12116)
Supplement: Table S1 — Details of differentially expressed genes in the ‘neurological disorders’ functional pathway identified in IPA. Table S2. Details of differentially expressed genes in the ‘inflammation’ functional pathway identified in IPA. Table S3. Genes from the microarray chosen for quantitative validation with qRT-PCR, the reasons and data obtained from qRT-PCR analysis. Numbers are the mean difference (± the standard error of the mean) in CT values between the gene of interest and the house keeper gene GAPDH for SHRSP and WKY at ages 5–21 weeks and from frontal (F) and mid-coronal (M) brain sections. [file nan0040-0855-SD1.pdf]

SUPPLEMENTARY MATERIAL

Supplementary Table 1: Details of differentially expressed genes in the “neurological disorders” functional pathway identified in IPA.

Functions Annotation	p-Value	Predicted Activation State	Regulation z-score	Molecules	# Molecules
Encephalopathy	1.51E-10		-0.706	ALB, BHLHE40, C20orf7, C3, C4B (includes others), CTGF, DUSP1, EGR1, EGR2, EGR4, FAM173A, FGF12, FGF13, FKBP8, FOS, FOXG1, GABRA5, GFAP, GOLPH3, GPR98, HLA-DMA, IER5, JUNB, KCNC2, MAL2, MAP1B, MAP4K1, MYO1B, MYT1L, NFIA, NFIB, NGFR, PDCL, PDE10A, PGRMC1, PLCB1, PLP1 (includes EG:18823), POLL, POLR2I, PTEN, PTGS2, S100B, SCN2A, SCN3A, SCOC, SERPINI1, SGK1, SLC1A3, SNAP25, SSR3, STK17B, VSNL1, ZNF440/ZNF808	53
Huntington's disease	1.92E-09			BHLHE40, C3, C4B (includes others), CTGF, EGR1, EGR2, EGR4, FAM173A, FGF12, FGF13, FOS, FOXG1, GABRA5, GFAP, IER5, JUNB, MAL2, MYO1B, MYT1L, PDCL, PDE10A, PGRMC1, PLCB1, POLR2I, SCN2A, SCN3A, SCOC, SERPINI1, SGK1, SLC1A3, SNAP25, SSR3, VSNL1, ZNF440/ZNF808	34
Neuromuscular disease	2.95E-08			ALB, BHLHE40, C3, C4B (includes others), COLQ, CTGF, EGR1, EGR2, EGR4, FAM173A, FGF12, FGF13, FOS, FOXG1, GABRA5, GFAP, HLA-DMA, IER5, JUNB, MAL2, MAP1B, MYO1B, MYT1L, NGFR, PDCL, PDE10A, PGRMC1, PLCB1, POLR2I, PTGS2, S100B, SCN2A, SCN3A, SCOC, SERPINI1, SGK1, SLC1A3, SNAP25, SSR3, VSNL1, ZNF440/ZNF808	41
Movement disorder	3.96E-07			BHLHE40, C3, C4B (includes others), CTGF, EGR1, EGR2, EGR4, FAM173A, FGF12, FGF13, FOS, FOXG1, GABRA5, GFAP, IER5, JUNB, KCNC2, MAL2, MAP1B, MYO1B, MYT1L, NGFR, PDCL, PDE10A, PGRMC1, PLCB1, PLP1 (includes EG:18823), POLR2I, PTGS2, SCN2A, SCN3A, SCOC, SERPINI1, SGK1, SLC1A3, SNAP25, SSR3, VSNL1, ZNF440/ZNF808	39
Seizures	8.58E-06	Increased	2.135	BHLHE40, C3, FOS, GABRA5, GJC2, GPR98, KCNC2, MAP1B, NGFR, PLCB1, PLCL1, PTEN, PTGS2, SCN2A, SLC1A3, TRIM3	16
Multiple system atrophy	5.70E-04			C20orf7, SCN2A, SCN3A, SLC1A3	4

Pelizaeus-Merzbacher disease	1.22E-03	GJC2, PLP1 (includes EG:18823)	2
Major depression	1.78E-03	BTG2, C7orf23, GABRA5, GFAP, IGFBP2, PDE10A, PSIP1, SYMPK, TTR	9
Stroke	1.95E-03	ALB, GABRA5, NGFR, PTGS2, S100B, SNAP25, VSNL1	7
Leakage of blood-brain barrier	2.01E-03	PTGS2, SERPINI1	2
Allodynia	2.34E-03	PTGDS, PTGS2, S100B, SLC1A3	4
Agenesis of corpus callosum	4.15E-03	MAP1B, NFIB	2
Hyperproliferation of brain cells	4.15E-03	BTG2, SSTR2	2
Cerebellar ataxia	4.24E-03	SCN2A, SCN3A, SLC1A3	3
Fragile X syndrome	4.24E-03	SCN2A, SCN3A, SLC1A3	3
Amyotrophic lateral sclerosis	4.73E-03	GABRA5, GFAP, NGFR, PTGS2, SCN2A, SCN3A, SLC1A3	7
Cervical spondylotic myelopathy	4.80E-03	SCN2A, SCN3A, SLC1A3	3
Tumorigenesis of brain cells	5.48E-03	MYCN, SSTR2	2
Neurodegenerative disorder	7.88E-03	C20orf7, C3, CTGF, GABRA5, GFAP, GJC2, IGF2, IGFBP2, IGFBP6, NGFR, PLP1 (includes EG:18823), PTEN, PTGDS, PTGS2, SCN2A, SCN3A, SLC1A3, TTR	18
Schizophrenia	8.64E-03	EGR4, GABRA5, GFAP, GNAI1, MLLT11, NFKBIA, NPTN, PLP1 (includes EG:18823), PNPLA8, PTGS2, S100B, SERPINI1, SLC1A3, SNAP25, TTR, VSNL1	16
Age-related macular degeneration	9.89E-03	C2, C3, CFH	3
Progressive supranuclear palsy	9.89E-03	SCN2A, SCN3A, SLC1A3	3
Macrocephaly	1.05E-02	POLL, PTEN	2
Brain cancer	1.25E-02	BTG2, FOXG1, GFAP, IGF2, IGFBP2, MCL1, MMP14, MYCN, PTEN	9
Hydrocephalus	1.29E-02	GFAP, NFIA, POLL, S100B	4

Neuropathy	1.40E-02	EGR1, EGR2, GABRA5, GFAP, HLA-DMA, MAP1B, NGFR, PLP1 (includes EG:18823), PTGS2, SCN2A, SCN3A, SGK1, SLC1A3, SNAP25, TTR	15
Cockayne syndrome type I	1.44E-02	ERCC8	1
Lhermitte-Duclos disease	1.44E-02	PTEN	1
Macrocephal-autism syndrome	1.44E-02	PTEN	1
Pelizaeus-Merzbacher-like disease	1.44E-02	GJC2	1
Pitt-Hopkins syndrome	1.44E-02	TCF4	1

Supplementary Table 2: Details of differentially expressed genes in the “inflammation” functional pathway identified in IPA.

**Functions Annotation**	**p-Value**	**Predicted Activation State**	**Regulation z-score**	**Molecules**	**# Molecules**
Rheumatic disease	3.82E-06		0.071	ALB, C2, C3, C4B (includes others), CLIC2, COL3A1, COL6A1, CPE (includes EG:12876), DUSP1, EGR2, FOS, GABRA5, GALNT2, GOLPH3, HLA-C, HLA-MA, MAP4K1, MAPRE1, MCL1, MMP14, NFKBIA, NGFR, NR4A3, PHACTR3, PTEN, PTGDS, PTGS2, RALB, RNF149, RNF39, RPS16, RPS18, SCN2A, SCN3A, SLC1A3, SNAP25, STK17B, TCF4, VPS52 (includes EG:224705)	39
Arthritis	1.32E-05		0.449	ALB, C2, C3, C4B (includes others), CLIC2, COL3A1, COL6A1, CPE (includes EG:12876), DUSP1, FOS, GABRA5, GALNT2, GOLPH3, HLA-C, HLA-DMA, MAP4K1, MAPRE1, MCL1, MMP14, NFKBIA, NGFR, NR4A3, PHACTR3, PTEN, PTGDS, PTGS2, RALB, RNF149, RNF39, RPS16, RPS18, SNAP25, STK17B, TCF4, VPS52 (includes EG:224705)	35
Rheumatoid arthritis	7.55E-05			ALB, C2, C4B (includes others), CLIC2, COL3A1, DUSP1, FOS, GABRA5, GALNT2, GOLPH3, HLA-C, HLA-DMA, MAPRE1, MCL1, NFKBIA, NGFR, NR4A3, PHACTR3, PTGS2, RALB, RNF149, RNF39, RPS16, RPS18, STK17B, TCF4, VPS52 (includes EG:224705)	27
Juvenile rheumatoid arthritis	3.11E-04			ALB, CLIC2, FOS, MCL1, NFKBIA, NR4A3, PTGS2, RALB	8
Polyarticular juvenile rheumatoid arthritis	1.61E-03			FOS, MCL1, NFKBIA, NR4A3, PTGS2, RALB	6
Splenomegaly	3.82E-03		-0.417	C3, C4B (includes others), INPP5D, NFKBIA, PTEN	5
Pulmonary interstitial fibrosis	4.15E-03			EGR1, PTGS2	2
Cervical spondylotic myelopathy	4.80E-03			SCN2A, SCN3A, SLC1A3	3
Acne	1.11E-02			COL3A1, CYP11B1, PLA2G2A, PTGS2, SGK1	5
Lichen planus	1.43E-02			COL3A1, HBB, HLA-C, IFI27, IGFBP6	5
Allergic contact eczema	1.44E-02			HLA-C	1
Arthritis of bone	1.44E-02			MMP14	1
Chronic suppurative sinusitis	1.44E-02			POLL	1
